# Flexible and Superhydrophobic Silver Nanoparticles Decorated Aligned Silver Nanowires Films as Surface-Enhanced Raman Scattering Substrates

**DOI:** 10.1186/s11671-019-3117-5

**Published:** 2019-08-22

**Authors:** Jianchao Wang, Guobin Yi

**Affiliations:** 1 The Collaborative Innovation Center of Manganese-Zinc-Vanadium Industrial Technology, National Demonstration Center for Experimental Chemistry Education, Hunan Engineering Laboratory for Analyse and Drugs Development of Ethnomedicine in Wuling Mountains, Jishou University, Jishou, 416000 China; 2 School of Chemical Engineering and Light Industry, Guangdong University of Technology, Guangzhou, 510006 Guangdong China

**Keywords:** Superhydrophobic, Flexible, Surface-enhanced Raman scattering, Plasmonic, Self-assembly, Silver nanowires

## Abstract

Flexible and superhydrophobic silver nanoparticles decorated aligned silver nanowires (AgNWs@AgNPs) films were employed as efficient surface-enhanced Raman scattering (SERS) substrates to investigate the SERS properties of the Rhodamine B (RB). Aligned silver nanowires were fabricated via interface self-assembly technique and incorporated into shape memory polyurethane (SMPU) by hot-press method, which not only endow the composites with ordered array characteristics but also flexibility due to the presence of polymer. After an electrochemical deposition combined with a galvanic reaction, AgNWs@AgNPs was obtained. At last, the substrate was functioned with perfluorodecanethiol (PFDT), and the target flexible and superhydrophobic silver nanoparticles decorated aligned silver nanowires substrate was obtained. The substrate confines water droplet in a small area, and the analytes were enriched owing to the concentrating effect. The SERS assay using the as-synthesized flexible and superhydrophobic silver films as substrates can detect Rhodamine B as low as10^−10^ M. The mechanism is thought to relate to the formation of robust superhydrophobic film, which is based on micro- and nanoscaled hierarchical structure provided by the AgNWs@AgNPs layer, strong adhesion between the SMPU film and the AgNWs@AgNPs layer, and the low surface energy molecule adsorption on the silver surface. The combined superhydrophobic and flexible properties endow the SERS substrate with improved detection limit for practical SERS applications.

## Introduction

Surface-enhanced Raman scattering (SERS) is recognized as an unprecedented technique that could be used for ultra-high sensitive detection of trace or even single-molecule [1–4]. Over the past several decades, much attention has been paid to novel SERS substrates fabrication and their applications to biomedicine and environmental analysis. The enhancement mechanism of SERS has been mainly ascribed to electromagnetic field enhancement. Regions of concentrated fields, the so-called hot spots, usually located at the gaps between particles, sharp tips, and high curvature points were crucial for high-sensitivity surface-enhanced spectroscopy [5–7]. The Raman signal of probe molecule in the vicinity of plasmonic structure could be enhanced as high as |E|^4^ [8]. Metal nanostructures with giant electric field due to localized surface plasmon resonances have been widely applied in SERS detection. Highly efficient SERS substrates, ranging from colloidal metal nanoparticles [9], roughened electrodes [10], metal films produced by vacuum deposition [11] to plasmonic nanoarray on planar substrates [12], benefit from the development of nanoscience and technology. The former two systems are low cost and easy to produce with poorly controllable fabrication processes; the latter plasmonic structures fabricated by top-down possess high signal enhancement and reproducibility. In this way, plasmonic structures could be fabricated to any desired configuration to meet the requirements for ultrasensitive SERS assay but require complex fabrication processes. Self-assembly [13, 14] is an effective approach to organize well-ordered nanostructures from various nanoparticles with controllable interparticle spacing, and avoid problems from lithography method, such as high cost, low yield, the complex processing procedures, and the dependence on special equipments. Significant progress has been made on the preparation of aligned silver nanowires films by self-assembly.

Superhydrophobic surfaces are usually made by controlling surface roughness of various materials and the surface chemical properties [15]. There are mainly two methods to prepare superhydrophobic SERS platform. The first is deposition of a thin film of metal on superhydrophobic surface, such as lotus and rose-petal-like surfaces, imparting plasmonic properties to the superhydrophobic surface [4, 16]. The second is that plasmonic hierarchical micro- and nanostructures was functioned by low surface energy coatings [17–19]. Superhydrophobic SERS substrate not only provides SERS hot spots but also enriches analyte molecules in a small area preventing the sample from spreading. Lee [20] assembled Ag nanocubes using the Langmuir-Blodgett as plasmonic nanostructures to fabricate superhydrophobic SERS platform. The superhydrophobicity of the substrate can be used for analyte concentration and trace detection [16]. The nanoparticles were easily detached from substrate because of the weak physical adsorption on the surface of silver. To firmly fix the nanoparticles, Hasell [21] took advantage of the physical constraint of the polymer template to fix nanoparticles. After coating a small layer of polymer, the assembled Ag nanocubes are more stable, but the “coating” process by the additional layer of polymer reduces the surface roughness that is bad for increasing surface roughness. Thus the fabrication of uniform nanostructures with stable, large surface roughness, and easily to fabricate is still a challenge.

Conventional rigid substrates are nonportable and unsuitable for practical samples. While, flexible substrates offer advantages in that it can be wrapped around non-planar substrates, or used as swabs to collect samples [22]. Moreover, it can be easily tailored into any desired shape or size. Therefore, the flexibility substrate, with high detective sensitivity, may be prospective in real-world SERS applications. Flexible SERS substrate is composed of plasmonic nanostructure which is incorporated into flexible materials such as paper [14], cotton [23], carbon nanotubes [24], graphene [25], and polymer materials [26]. Martín [27] reported flexible ordered vertical Au nanorod arrays and the detection limit was 5 nM using crystal violet (CV) as the detecting probe. Mekonnen [14] used Ag@SiO_2_ nanocube-loaded miniaturized filter paper as SERS substrate to detect melamine with a limit of detection of 0.06 mg L^−1^. He [28] fabricated Ag dimers and aligned aggregates which are assembled within poly(vinyl alcohol) nanofibers via electrospinning technique. The Ag/PVA nanofiber platform could detect as low as 10^−6^ M using 4-MBA probe molecule. Park [29] demonstrates transparent and flexible SERS substrates on a polydimethylsiloxane film embedded with gold nanostar and achieve a trace amount of benzenethiol (10^−8^ M) detection.

Shape memory polyurethane (SMPU) is a smart material that shows great potential in mechanical, optical properties, and tailorability. Compared with other flexible substrates (such as paper, PVA, rubber, and so on), it has superiority for the following reasons. Firstly, SMPU exhibits shape memory effect. SMPU could memorize its original shape or state to avoid irreversible plastic deformation [30]. Secondly, the gap between adjacent plasmonic structures is one of the most significant factors for SERS responses. Particle separation can be optimized by mechanically manipulating the stretchable substrate to vary the gap distance thereby changing SERS signal. SMPU is expected to be a good candidate to be used as the assistant material for smart SERS substrates.

In this paper, we report very promising SERS platforms based on flexible superhydrophobic films comprised of aligned AgNWs-AgNPs monolayer. We applied the as-prepared SERS platform for sensitive SERS detection of Rhodamine B (RB) and found that the SERS signal can be significantly improved. The detection limit could be as low as 10^−10^ M for Rhodamine B. The as-prepared flexible and superhydrophobic platforms will find promising practical SERS applications.

## Methods

### Reagents

1H, 1H, 2H, 2H-perfluorodecanethiol (PFDT) were purchased from Sigma-Aldrich. AgNO_3_ and CuSO_4_ (analytical grade) were obtained from Beijing chemical reagents company. Silver nanowires aqueous suspension (diameter 300 nm, length 30 μm) was purchased from Haoxi research nanomaterials, Inc. Non-crystalline SMPU was synthesized [31].

### Fabrication of Aligned Silver Nanowires Films

The aligned silver nanowires (AgNWs) films were prepared by interfacial assembly method [32]. Briefly, AgNWs aqueous suspension (5 mg/mL) was added onto the liquid surface of chloroform. Subsequently, acetone was added dropwise to the AgNWs suspension. A few minutes later, aligned AgNWs films was achieved on the surface of aqueous phase until a sparkling mirror-like surface emerged. The ordered AgNWs film was then transferred onto precleaned chips. The aligned AgNWs-SMPU composite films were prepared by hot-press method and labeled as S0.

### Fabrication of Cu-Decorated Aligned AgNWs Films

The aligned AgNWs film was immersed in a mixture of aqueous copper sulphate solution (70 g/L), sulfuric acid 200 g/L, hydrochloric acid (50 ppm), Bis-(3-sodiumsulfopropyl disulfide) 1 ppm, polyethylene glycol 6000, and Janus Green (1 ppm) for electrochemical deposition of copper film at 0.1 A via two-electrode system. A copper plate and the aligned AgNWs films were used as anode and cathode, respectively. The deposition was carried out for a certain time at room temperature, and the time is 5, 15, 30, and 60 s, respectively. After rinsing with deionized water, and N_2_ drying, the Cu-decorated–AgNWs films were obtained and labeled as S1, S2, S3, and S4.

### Fabrication of AgNWs@AgNPs Films

The Cu-decorated AgNWs film was immersed in an aqueous AgNO_3_ solution (1 × 10^−3^ M) for 1 min to form silver nanoparticles (AgNPs) by a galvanic reaction between Cu^0^ and Ag^+^ ions. After rinsing with deionized water, and N_2_ drying, the silver nanoparticles decorated aligned silver nanowires (AgNWs@AgNPs) film was obtained.

### Superhydrophobic AgNWs@AgNPs Films

The AgNWs@AgNPs film was immersed in a 5 mM PFDT in 1:1 ethanol/hexane solution for 15 h to deposit a layer of PFDT on the surface of the AgNPs and AgNWs. The superhydrophobic AgNWs@AgNPs films were washed with ethanol repeatedly and dried prior to the measurements.

### Characterization

The sample was characterized by scanning electron microscope (SEM) (JEOL, JSM-7001F, Japan), UV–vis spectrophotometer (UV 2450, Shimadzu), X-ray diffraction (XRD) (X’Pert Powder, Holland) with Cu-Kα1 line (λ = 0.1540 nm), and Cu-Kα2 line (0.1544 nm) in the Bragg angle ranging between 30° and 90°. DC power supply (Zhaoxin Electronic, Shenzhen, China) (RXN-605D) was used in sample fabrication. Static water contact angle measurement was carried out by contact angle goniometer (JC2000D1, Shanghai, China) at five positions on each substrate using a drop of water (5 μL). The static contact angle is abbreviated as CA throughout the text. The Raman spectra were collected using a Raman spectroscopy (Raman, HORIBA Jobin Yvon LabRAM HR 800, France) with the excitation wavelength 633 nm, the maximum excitation power 1.7 mW, integration times 20 s, and spot diameter of laser beam about 1 μm.

## Results and Discussion

**Fig. 1:**
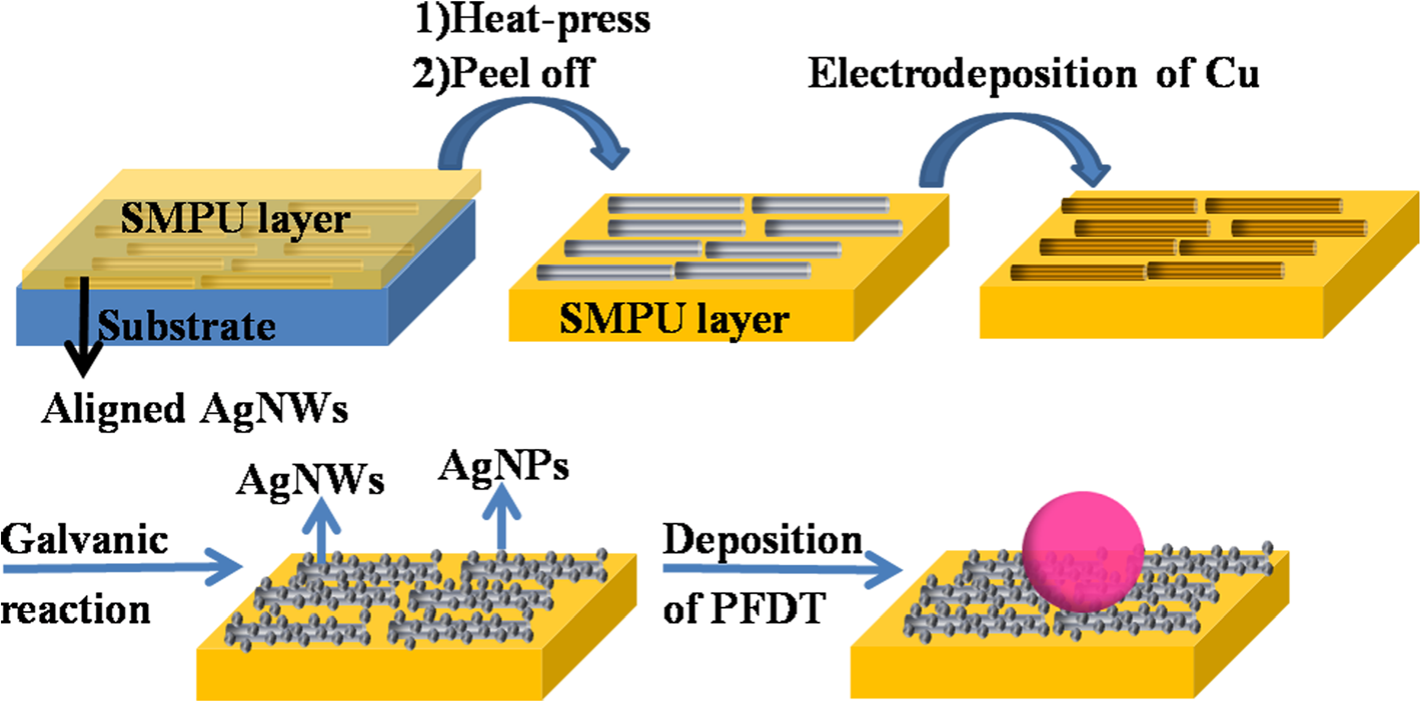
Schematic illustration of the fabrication process for flexible and superhydrophobic AgNWs@AgNPs films. The concentrating effect of the superhydrophobic substrates, and the prepared flexible substrate and the SMPU film

The process for fabricating superhydrophobic films is schematically illustrated in Fig. 1. The fabrication process included three steps, containing preparation of flexible substrate, surface roughening, and surface hydrophobization. The specific process is as follows: (1) the interfacial assembly process was employed to fabricate aligned AgNWs film. Aligned AgNWs film was incorporated into SMPU substrate via hot-press treatment. (2) A layer of copper was deposited on the surface of AgNWs by an electrochemical deposition process via two-electrode system, which is controlled by adjusting the deposition time. Silver nanoparticles (AgNPs) were deposited on the surface of the AgNWs by galvanic displacement between Cu^0^ and Ag^+^ ions, (3) followed by hydrophobization with 1H, 1H, 2H, 2H-perfluorodecanethiol (PFDT).

**Fig. 2:**
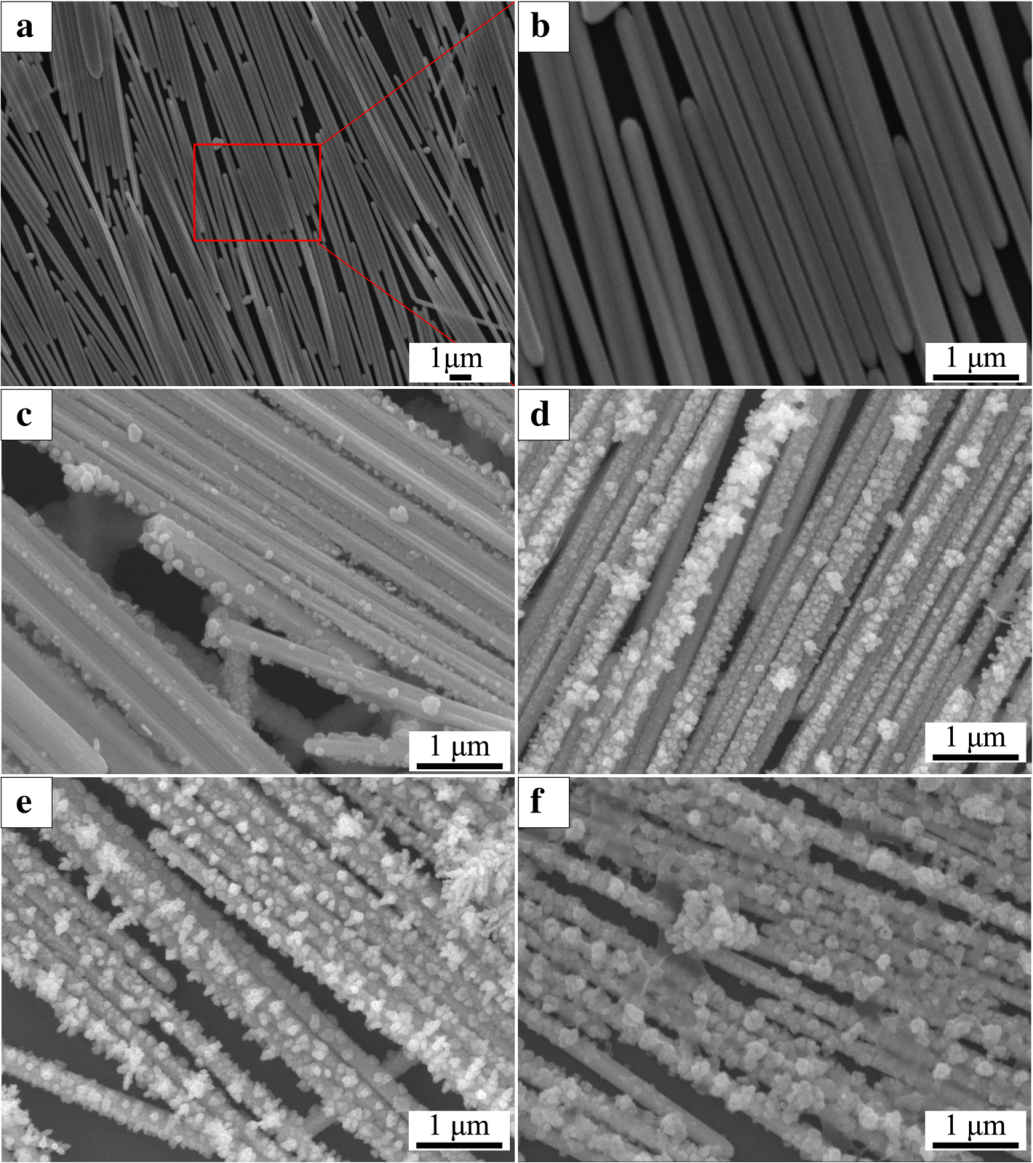
SEM images of the aligned AgNWs films and different AgNWs@AgNPs films. **a**, **b** Different magnifications of SEM images of aligned AgNWs film. **c**–**f** Different AgNWs@AgNPs-1, 2, 3, 4 films standing for the deposition time 5 s, 15 s, 30 s, 60 s respectively

SEM images in Fig. 2a, b show that the AgNWs with smooth surface are aligned parallel to each other, forming a close contact, highly arrayed monolayer with some large intervals and multilayer structures formed during transfer process. Figure 2c–f shows that AgNPs are formed on the surface of the AgNWs film. The size and the distribution of nanoparticles increased with the electrodeposition time increased from 5 to 60 s. It is suggested that the particle size can be tuned by changing deposition time. The thickness of SMPU used in our flexible SERS platform is about 50 μm. The largest SERS enhancement often presents at the junction between coupled nanometer-sized objects. Calculations have shown that the interstitial gaps between nanoparticles separated by 1 nm can provide an enhancement factor of 10^10^ [8]. Furthermore, the SERS mapping image of the coupled roughened silver nanowires and the coupled smooth silver nanowires show significant difference in SERS intensity. The noticeable SERS intensity of the coupled smooth nanowires are mainly focused at the ends of the nanowires, whereas for the coupled roughened silver nanowires system, the hot spots are located at much wider region distribution areas including ends, gaps, and the whole surface of the roughened silver nanowires. The result gives favorable evidence for enhanced SERS signal of aligned AgNWs-AgNPs monolayer [33].

**Fig. 3:**
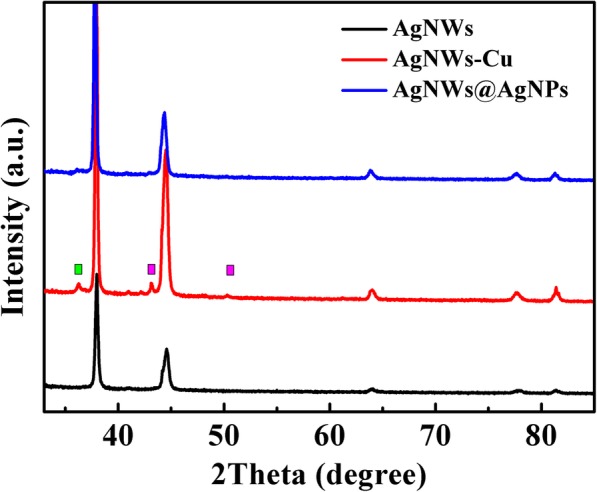
XRD pattern of the aligned AgNWs, Cu-decorated aligned AgNWs film, and AgNWs@AgNPs film

Figure 3 presents the XRD pattern of AgNWs film, Cu-decorated AgNWs film, and AgNWs AgNPs film. The diffraction pattern for the AgNWs film has four peaks at 36.41, 42.67, 62.93 and 75.91, corresponding to the (111), (200), (220), and (311) fraction direction of face centered cubic structure of silver (JCPDS No. 4-0783), respectively. For the Cu electrodeposited film, besides Ag peaks, additional peaks (purple squares) appeared at 43.15, and 50.36, which can be indexed to copper (JCPDS 04-0836) and peak (green square) at 36.28 can be indexed to silver oxide (JCPDS 19-1155). For AgNWs@AgNPs film, the diffraction peaks of copper (111) decreased sharply until completely disappeared. This showed galvanic displacement reaction took place. The diffraction peaks of Ag were sharp and intense, indicating their highly crystalline nature. No impurity peaks were observed, confirming the high purity of the samples.

**Fig. 4:**
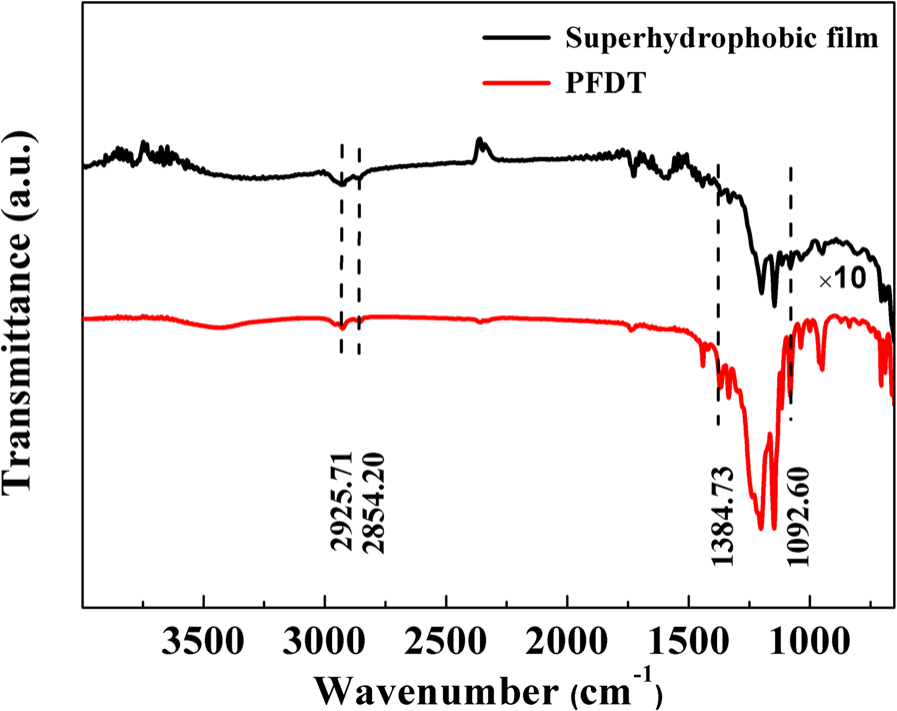
FT-IR spectra of PFDT and the superhydrophobic film (PFDT modified AgNWs@AgNPs films)

FT-IR spectra (Fig. 4)were carried out to demonstrate the adsorption of PFDT on the surface of substrates, and the results were shown in Fig. 4. The peaks at 2853 cm and 2925 cm could be ascribed to the symmetric and asymmetric CH vibrations while those peaks at 1092 cm and 1384 cm could be assigned to the symmetric and asymmetric CF vibrations. Compared with typical PFDT (2853, 2952, 1244, and 1354 cm ), some of these peaks were redshifted, suggesting that the surface is successfully modified with PFDT. The result indicated that PFDT was adsorbed on the silver surface and that the molecular plane was almost perpendicular to the surface. The vibration frequencies of CF shift toward a lower wave number suggested that PFDT formed an ordered monolayer on the surface [34].

### Static Contact Angles

**Fig. 5:**
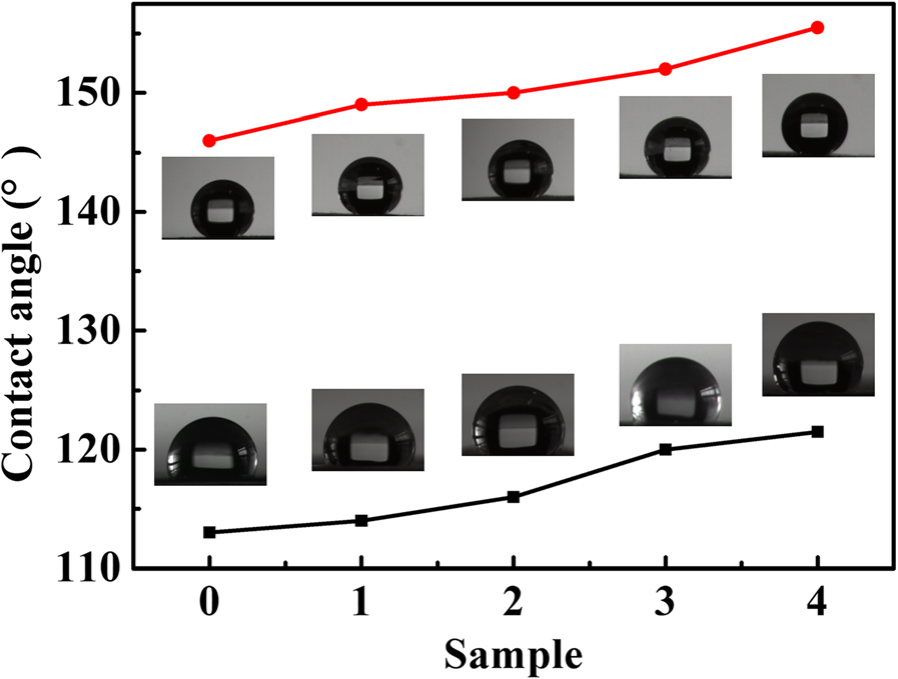
Contact angle images of water droplet on AgNWs (sample 0), and AgNWs@AgNPs − 1, 2, 3, 4 composite films (sample 1, 2, 3, 4) (black) and corresponding superhydrophobic films (red). The insets the corresponding water contact angles of the films

In order to evaluate hydrophobic effect of PFDT-modified AgNWs film composite, contact angle was examined. As shown in Fig. 5, AgNWs and AgNWs@AgNPs film has a water contact angle from 113° to 121°. After the deposition of PFDT on the surface of the AgNWs@AgNPs films, the contact angle significantly increased to 155°. The transition from hydrophilicity to superhydrophobicity can be attributed to the increase of roughness and reduction of surface free energy through chemical modification of AgNWs film surfaces. Increase in the deposition time results in more cracks and sharp edges formed on the surface of AgNWs, and existing voids could entrap air which is expected to favor surface hydrophobic properties, which also provide more plasmonically active surface area.

### Concentrating Effect

**Fig. 6:**
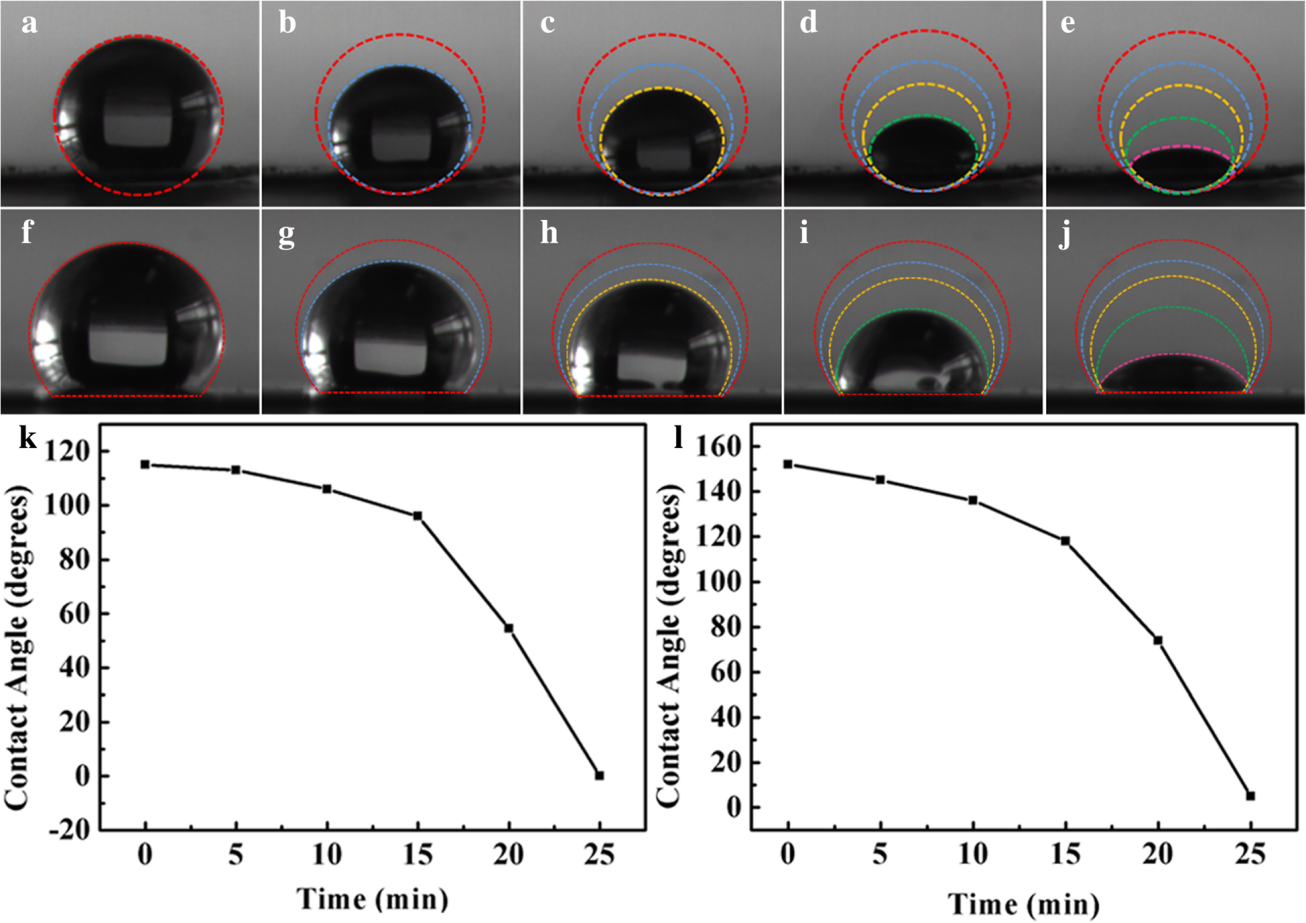
**a**–**e** Images of evaporation process of a droplet of RB aqueous solution dripped onto superhydrophobic surface. **f**–**j** Images of evaporation process of a droplet of RB aqueous solution dripped onto AgNWs@AgNPs surface. **k**, **l** Plot of contact angle with different evaporating times at 0, 5, 10, 15, 20 min on AgNWs@AgNPs and superhydrophobic surface

To investigate the concentrating effect of superhydrophobic substrate, water contact angles on superhydrophobic and AgNWs @AgNPs film as a function of evaporating time were studied. Figure 6a–e shows evaporation process of 5 μL droplet of RB aqueous solution on AgNWs@AgNPs film with duration of evaporation of 25 min. Figure 6f–j shows corresponding process on superhydrophobic substrate. It was found that the drop was reduced in volume, from large spherical shape to small spherical segment, and ultimately pinned to dried surface area. The solution therefore became more and more concentrated. After the complete evaporation of solvent, the solute was deposited in a confined region with an area of a few square microns. During evaporation, the solid liquid contact area was almost unchanged, and the three-phase contact line of droplets was stable. The result indicated that the size of the spot area was mainly determined by wettability of the substrate. The evaporation process was similar for superhydrophobic substrate, and the difference was that the contact area was much smaller indicating that concentrating effect was enhanced on superhydrophobic substrate.

Superhydrophobic substrate confines the solute in a small area compared to that of the AgNWs film surfaces [20]. After the drying of the droplets on two kinds of substrates, spot size of the droplet was examined. The results showed that area of spot was about 0.60 mm^2^ for superhydrophobic substrates, and 3.2 mm^2^ for the AgNWs@AgNPs film, which is five times bigger than the former one. These results demonstrate that our superhydrophobic surface was able to concentrate and direct liquid analyte into a small area to enhance analyte concentration.

Figure 6k, l depicts the relationship between water contact angles on two kinds of substrates and evaporating times. It was found that the water contact angle decreased with time. Different factors contribute to the result. The decreased of CA can attribute to the following factors. First, water droplets were dipped into the groove of silver micro/nanostructures by capillary action changing the contact between water droplets and substrate from heterogeneous contact to homogeneous contact. Second, the binding force is not strong enough that PFDT were desorbed from the substrate due to the strong interfacial tension between the substrate and the droplet, resulting in decrease of surface energy. Third, ligand exchange between RB and PFDT through strong force between RB and substrate bringing about destroying hydrophobic layer, and the analyte adsorbed to the surface of the layer showing good effectiveness in SERS detection.

**Fig. 7:**
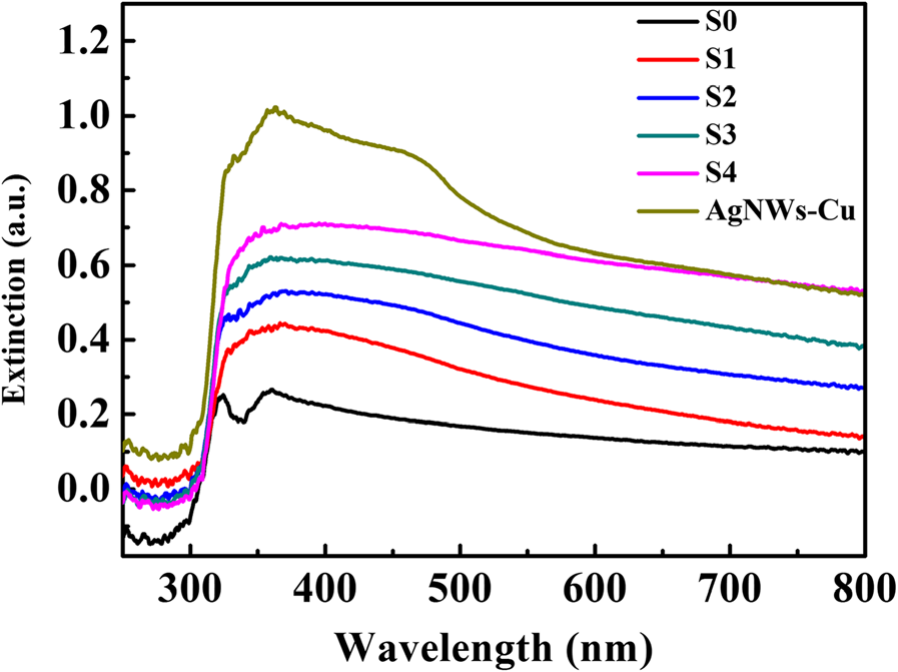
UV–vis extinction spectra of aligned Ag nanowire substrate (AgNWs), the Cu-decorated AgNWs film (AgNWs-Cu), and different silver nanoparticles decorated aligned silver nanowires films with different copper coating, the composite films were labeled as AgNWs@AgNPs-1, 2, 3, 4 respectively

Localized surface plasmon properties of plasmonic nanostructures is sensitive to the size, shape and dielectric environment of nanoparticles [35], and plays an important role in surface-enhanced Raman spectroscopy (SERS) applications. UV-Vis extinction spectra were used to investigate characteristic LSPR bands of AgNWs@AgNPs composite films. Figure 7 shows two characteristic peaks at 323, and 352 nm, which are optical characteristic of silver nanowires. After copper deposition, a broad absorption band at 280, and 570 nm appeared, which are ascribed to characteristics of copper film with an extensive pelectron delocalization, confirming the successful copper deposition. After the galvanic replacement between copper nanostructures and an AgNO3 solution, new absorption band at 450 nm appeared, which is attributed to the surface plasma resonance (SPR) of silver nanoparticles. With the increase of silver nanoparticles by extend plating time on Cu foil, the intensity of all the absorption peaks were enhanced with slight red-shift [36].

### Raman Analysis

**Fig. 8:**
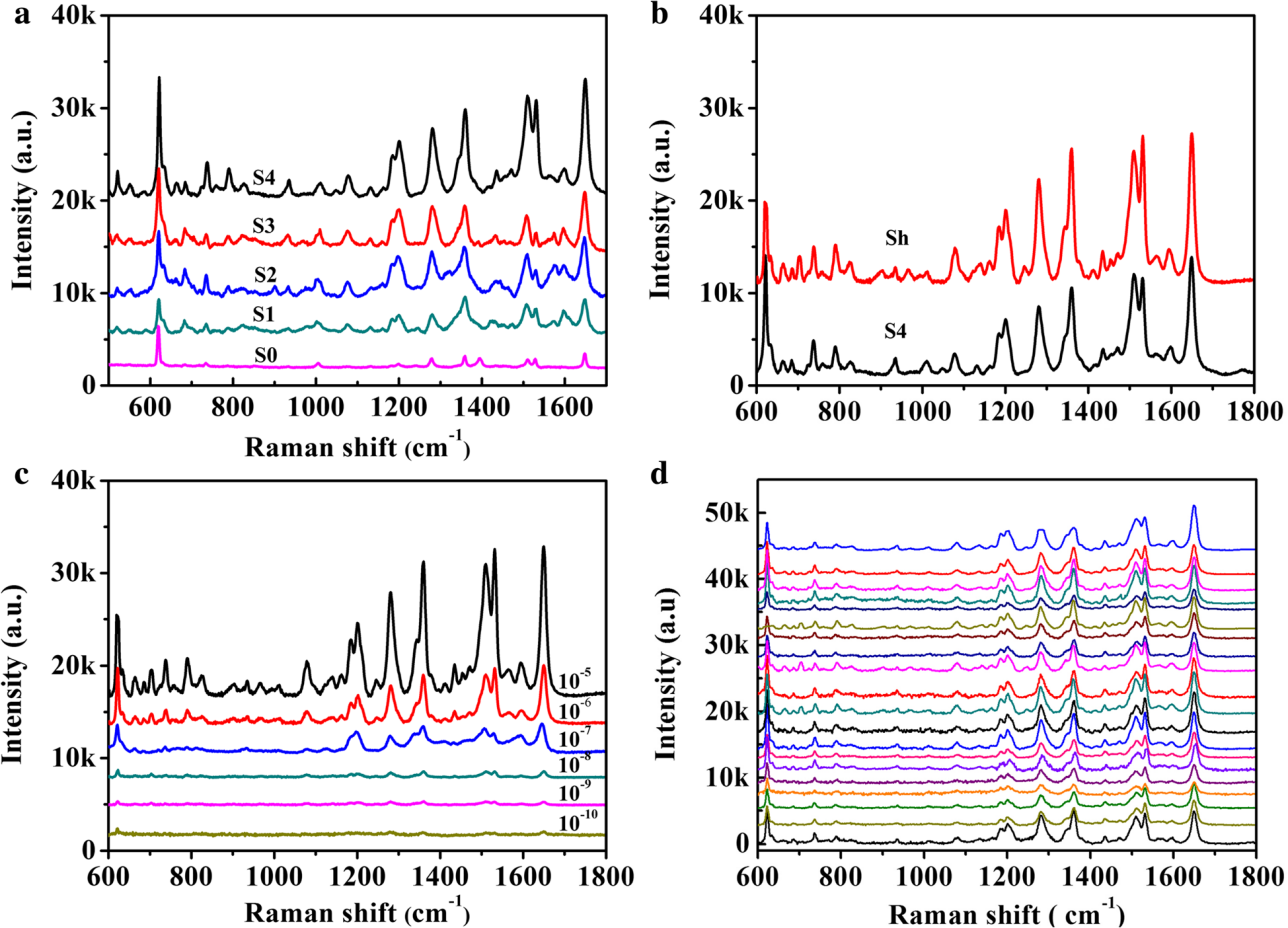
**a** SERS spectra of 10^−5^ M RB on different AgNWs@AgNPs substrates, S0, S1, S2, S3, S4. **b** SERS spectra of RB with two different substrates RB (10^−5^ M) (Sh: superhydrophobic counterpart of S4). **c** SERS spectra of RB at different concentration on flexible and superhydrophobic substrate. **d** Reproducibility of the SERS signals at twenty random sites (10^−6^ M)

SERS measurements were carried out to investigate the performance of AgNWs@AgNPs film and its superhydrophobic counterpart. A drop of RB solution (5 μL, 10^−5^ M) was added on substrates, and the corresponding spectra were collected in Fig. 8a. The Raman bands at 620 cm^−1^ is attributed to C-C-C stretch, and the peak at 1186 cm^−1^ corresponds to C-H in-plane bend, while the four peaks at 1280 cm^−1^, 1358 cm^−1^, 1506 cm^−1^, and 1650 cm^−1^ are designated to stretching vibration of aromatic C-C bond. The peak positions of different substrates were nearly the same and were coincident with the characteristic peaks of RB [37], and no obvious band shift was observed. The Raman signal from AgNWs@AgNPs film substrate increased dramatically with the increase of particle size. Surface plasma resonance (SPR) of metal nanoparticles plays an important role in enhancing SESR intensity. The reduction of gap distance of adjacent plasmonic nanostructure by decorating silver nanoparticles on the surface of nanowires have a significant effect on SERS response. The local EM field enhancement is amplified by coupling effects between adjacent nanoparticles. On the other hand, the SMPU could absorb water [38], resulting in a slight swell of the polymer and easy access of the probe molecules into the polymer and the hot spots which is essential for large Raman enhancement.

To investigate SERS responses of hydrophobic AgNWs@AgNPs film, we compared Raman intensity of RB on AgNWs@AgNPs film and hydrophobic counterpart, as shown in Fig. 8b. Further, 1.5-fold intensity enhancement could be achieved on the hydrophobicity substrate. It was supposed that the enhanced Raman intensity is mainly caused by concentrating effect. According to literature, SERS intensity enhancement shows a second-order dependence with respect to the decrease in spot diameter on a superhydrophobic substrate compared to a hydrophilic counterpart [39]. From above concentrating effect study, spot sizes on our superhydrophobic surfaces after natural evaporation of droplets is about five times smaller as compared to the spot sizes on a hydrophilic surface. The intensity enhancement is lower than the concentration factor of the hydrophobic substrate which may be related to the fact that not all RB molecules were adsorbed on the surface of the silver nanoparticles or nanowires since the existence of PFDT layer.

To test the limits of detection of the substrate, SERS spectra on the superhydrophobic substrates were measured after exposure to different concentrations of RB. Figure 8c shows that the SERS intensity increased with increasing concentration of the probe molecule. The characteristic Raman bands of RB at 1650 cm^−1^ still dominates, even at 10^−10^ M. At a lower concentration, the main feature of RB is comparable with background features from the SMPU, which are located at 868, 1468, and 1723 cm^−1^, respectively. However, weaker RB bands can still be identified. Such higher SERS activity of RB is expected to result in higher Raman scattering cross section of RB compared to SMPU and PFDT. Furthermore, the interaction of SMPU with the plasmonic nanostructure give rise to the formation of a direct Ag-N chemical bond, resulting in stronger interaction between amine group of RB and silver nanowires and nanoparticles. While for SMPU, silver nanowires were embedded into polymer substrate, physical interactions are dominant. Thus, the Raman signal of RB was more significant. The total Raman enhancement may be due to the double effect of concentrating and plasmonic coupling. Superhydrophobic substrates can confine analyte molecules into a smaller area, which was also the sensitive area of plasmonic nanostructures. The coincident make trace molecular detection possible. Moreover, no obvious peak was observed for PDFT, showing that the introduction of hydrophobic molecules did not affected Raman signals significantly. Therefore, the solution evaporation-induced concentrating process of superhydrophobic SERS platform render additional concentration increases to plasmonic nanostructures to further reduce the detection limit.

**Table 1:** Intensity statistics of the main peaks (1280 cm^−1^, 1506 cm^−1^, and 1650 cm^−1^) of Rhodamine B collected from 20 spots randomly

	I(1280)	I(1506)	I(1650)
Arithmetic mean	3026.94	3483.38	4576.80
Standard deviation (SD)	662.25	831.80	836.64
Relative standard deviation (RSD)	0.219	0.239	0.183

The uniformity of SERS substrate is one of the most important factors for quantitative detection. Twenty random positions were chosen to investigate uniformity of plasmonic nanostructure, and the representative results were showed in Fig. 8d using RB as a model molecule. Each band of the Raman spectrum exhibited remarkable uniformity. Through statistic on the most prominent band of 1280, 1560, and 1650 cm^−1^, the relative standard deviations are 21.9%, 23.9%, and 18.3% representatively, suggesting the uniformity of the prepared substrates (Table 1).

**Fig. 9:**
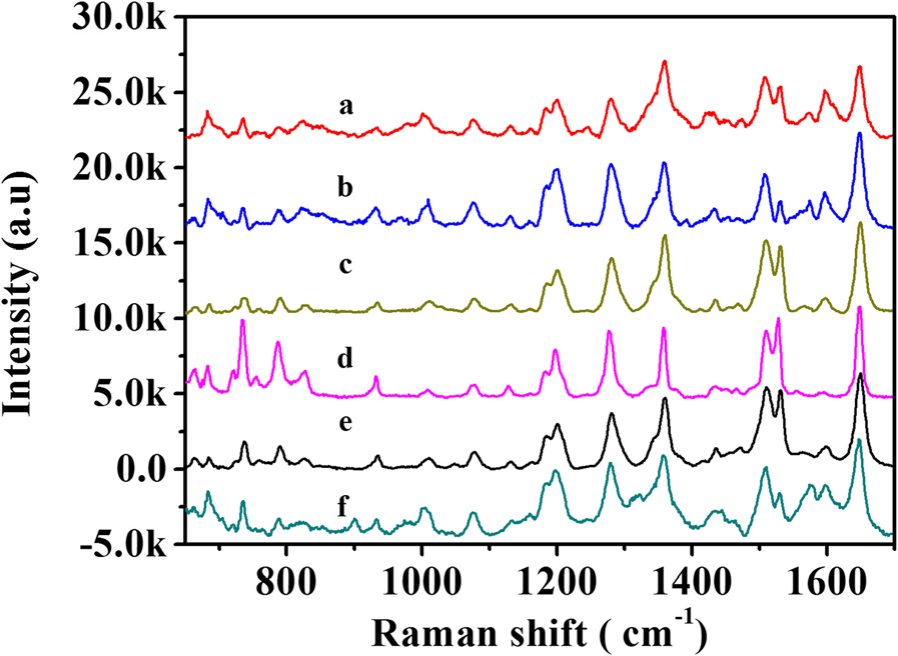
SERS spectra of 10^–6^ M RB on the flexible and superhydrophobic AgNWs@AgNPs substrate at different time-points (15 min, 30 min, 1 h, 2 h, 12 h, and 24 h)

For stability measurement, Rhodamine B (10^−6^ M) was employed as probe molecule. The results are presented in Fig. 9. From curves (a) to (f), we can see sharp characteristic peaks of Rhodamine B, which are obtained from substrates synthesized with different reaction time of 15 min, 30 min, 1 h, 2 h, 12 h, and 24 h. The most intense characteristic peaks appear at a Raman shift of about 1620 cm^−1^. We compared the height I_1650_ in order to evaluate the stability of SERS substrates according to their SERS efficiency. The results showed that the intensity of SERS has remained approximately constant during this period. Slight fluctuation of the peak intensity may be caused by inhomogeneity of the surface of the substrates. The results show that the self-assembled AgNWs@AgNPs substrates are stable, and they show the same performance after a day.

## Conclusion

In summary, we developed a method of preparation of superhydrophobic silver nanoparticles decorated aligned silver nanowires arrays on SMPU substrates that were employed as efficient substrates for SERS studies. Target substrates were fabricated by alignment of silver nanowires, decorating silver nanowires with silver nanoparticles, infusion into the polymer, and functionalization with PFDT. The resulting superhydrophobic substrate can confine water droplet of analyte molecules within a small area, combined with the enhanced electromagnetic field of plasmonic structures due to localized surface plasmon resonances; the sensitivity of detection was improved. Furthermore, the intensity was significantly enhanced with an increase in the contact angle. The detection limit was 10^−10^ M for Rhodamine B. The mechanism is based on the AgNWs@AgNPs layer provides micro- and nanoscaled hierarchical structures in support of superhydrophobicity, strong adhesion between the SMPU film and the AgNWs@AgNPs layer, and the hydrophobicity of film is successfully conveyed to the polymer based flexible layer. The combined superhydrophobic and flexible properties endow the SERS substrate with improved detection limit, sensitivity, and signal reproducibility for applying natural materials to practical SERS applications.

## Abbreviations

AgNPs: Silver nanoparticles
AgNWs: Silver nanowires
AgNWs@AgNPs: Silver nanoparticles decorated aligned silver nanowires
CA: Static contact angle
PFDT: Perfluorodecanethiol
RB: Rhodamine B
SEM: Scanning electron microscope
SERS: Surface-enhanced Raman scattering
SMPU: Shape memory polyurethane
XRD: X-ray diffraction
